# Role of cholinergic innervation in biliary remnants of patients with biliary atresia

**DOI:** 10.3389/fped.2023.1278978

**Published:** 2024-01-08

**Authors:** Jixin Yang, Xiaoqing Chen, Wenjing Wang, Yanwei Su, Keqin Liu, Adila Abudusalamu, Dandan Li, Ying He, Pusu Wang, Xiaofeng Xiong, Jiexiong Feng

**Affiliations:** ^1^ Department of Pediatric Surgery, Tongji Hospital, Tongji Medical College, Huazhong University of Science and Technology, Wuhan, Hubei Province, China; ^2^ Department of Pediatric Surgery, Jiangxi Provincial Children’s Hospital, Nanchang, Jiangxi, China; ^3^ School of Nursing, Tongji Medical College, Huazhong University of Science and Technology, Wuhan, Hubei Province, China

**Keywords:** cholangitis, biliary ductile, jaundice, acetylcholinesterase, parasympathetic nerve

## Abstract

**Objective:**

Biliary innervation is considered important in regulating the function of bile ducts, whereas the role of innervation in the hepatobiliary system of patients with biliary atresia (BA) remains unknown. This current study aims to investigate the role of innervation in biliary remnants and analyze the relationship between the innervation and prognosis of BA after surgery.

**Methods:**

Eighty-seven patients with type III BA who underwent the Kasai procedure were consecutively enrolled from January 2017 to September 2020. Innervation and ductules in remnants were examined by pathologists. Liver function, onset of cholangitis, jaundice clearance, and survival with the native liver were recorded. Patients were followed up for 24 months. The relationship between innervation and prognosis was analyzed.

**Results:**

In total, 67 patients had bile drainage postoperatively, and 21 biliary remnants contained neuronal plexuses where there was no neuron but nerve fiber bundles. Acetylcholinesterase staining was positive in all plexuses. In patients with bile drainage, those with plexuses had improved postoperative liver function, significantly better jaundice clearance 3 or 6 months postoperatively (50.0% vs. 19.1%, or 90.0% vs. 63.8%, respectively), fewer episodes of early cholangitis (10.0% vs. 34.0%), and better survival (80.0% vs. 61.7%) compared to those without. In addition, a larger area of plexuses was associated with a larger area of ductules (*R*^2 ^= 0.786, *p* = 0.000), less frequent (*p* = 0.000) and later cholangitis onset (*p* = 0.012), and better jaundice clearance (*p* = 0.063).

**Conclusions:**

Increased cholinergic innervation in biliary remnants may help reduce the onset of cholangitis and lead to better and earlier jaundice clearance. Thus, it improves the postoperative prognosis of patients with BA.

## Introduction

It is known that sympathetic and parasympathetic nerves exist around the intrahepatic and extrahepatic bile ducts (1). The parasympathetic nerve derives from the vagus nerve. Its anterior plexus surrounds the hepatic artery, and its posterior plexus surrounds the portal vein and bile duct. Research studies showed that the vagus nerve branches connect the liver and bile duct plexus and are distributed to each segment of the liver and around the bile ducts (2). This nerve distribution plays a vital role in regulating the functions of bile ducts, including promoting cholangiocyte proliferation and reducing apoptosis following bile duct injury (3). However, until now, there have been very few reports on the role of innervation in the hepatobiliary system in patients with biliary atresia (BA).

**Figure 1:**
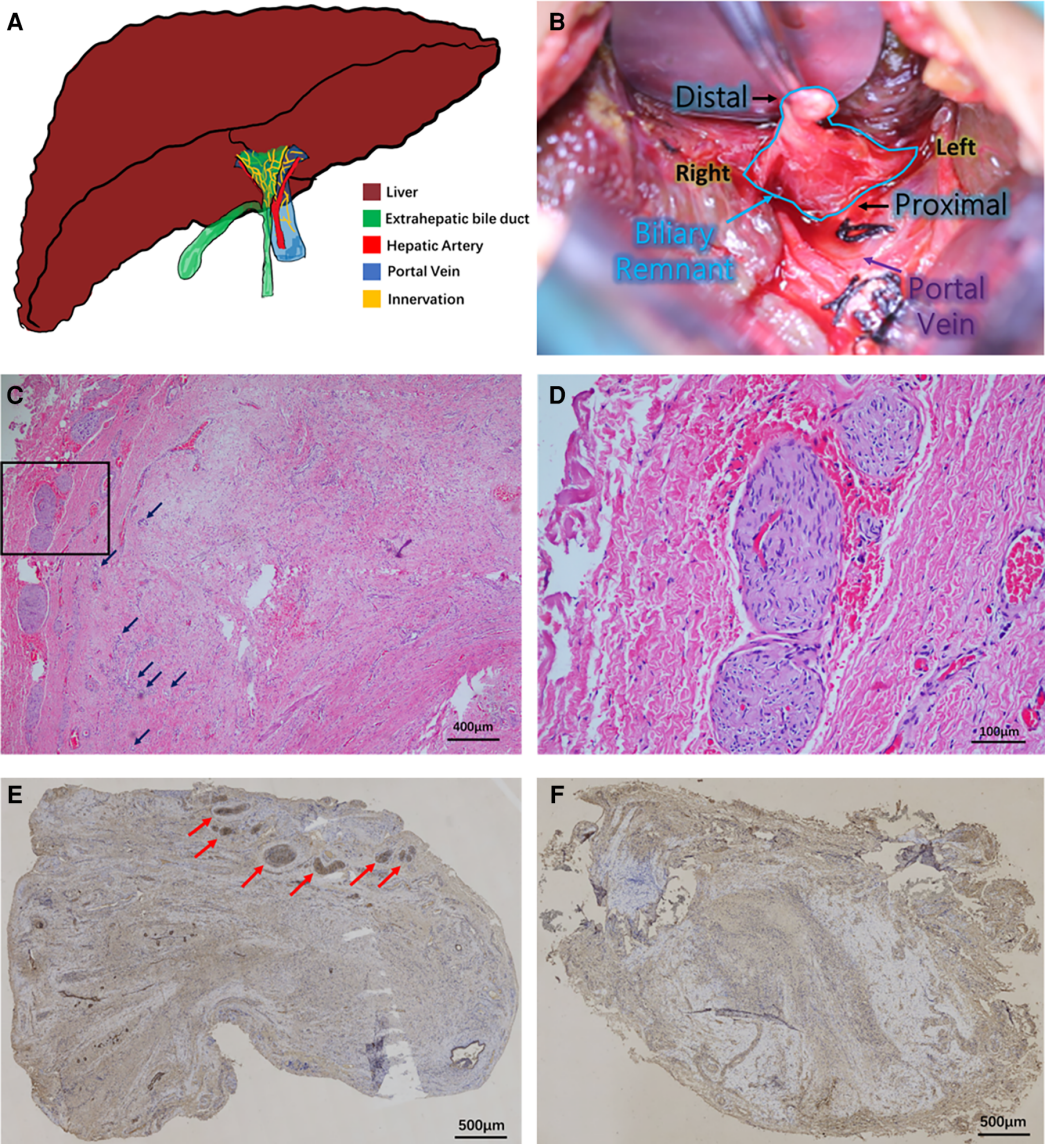
Innervation in biliary remnants. (**A**) Illustration of the hepatobiliary system in patients with biliary atresia. The atresia of the extrahepatic biliary system (green) is visible outside the fibrotic liver (brown). The right hepatic artery (red) and portal vein (blue) are behind the biliary remnants. Innervation (yellow) is present in the bile ducts and vascular system. (**B**) Intraoperative view of a biliary remnant in the Kasai procedure. The triangular remnant is stripped to the depth above the portal vein bifurcation and enlarged to the outer edges of the portal vein. (**C**) Hematoxylin–eosin staining of biliary remnants and neuronal plexuses. Growth of giant plexuses was observed in the marginal area of remnants of some cases. Some ductules were noted in the remnant (black arrow). The black frame area was further magnified in **(D**). Magnification: ×100. (**D**) Details of plexuses. Larger magnification of plexuses showed no neurons in the bundles but only thick nerve fiber bundles. The bundles were “wavy” or “vortex”-shaped. Magnification: ×400. (**E**) Acetylcholinesterase staining of plexuses. The plexuses were all positive, shown by immunohistochemical staining (red arrow). Magnification: ×40. (**F**) Biliary remnant without plexuses. No plexus was observed in some other cases. Magnification: ×40.

BA is the most common cause of obstructive jaundice in newborns (2). Patients may develop hepatic fibrosis 1 month after birth, and if untreated, the patients may die around 1 year of age (4). After the diagnosis of BA, the primary surgical treatment mainly involves Kasai portoenterostomy (KP). Although many factors affect the survival after KP, the favorable postoperative bile drainage and the onset of cholangitis are recognized as the most important determinants (5). In recent years, our studies have focused on the distribution of ductules in biliary remnants of patients with BA, and we observed that the number/area of ductules yielded by technical precision is closely related to effective bile drainage, jaundice clearance, and the first onset of cholangitis in patients after KP (6). When observing the ductules in biliary remnants, we noticed nerve plexuses in the biliary remnants of some BA patients in the meantime (Figure 1A). So far, there has not been any research focusing on the innervation in biliary remnants, but an early clinical investigation by Iwami et al. reported the abnormal proliferation of nerve fibers in the livers of patients with BA (7). They concluded that the innervation could be associated with immaturity or malformation in the biliary system. However, following this conclusion, they did not analyze the clinical significance or further discuss the possible mechanism of innervation. To understand the features and clinical significance of innervation in biliary remnants, we conducted this clinical study to reveal the characteristics of innervation and its impact at different postoperative stages after KP.

## Patients and methods

From January 1, 2017, to September 30, 2020, 87 patients with BA who underwent KP at our department were consecutively enrolled. This study was approved by the Institutional Review Board, and informed consent forms were signed. All patients were diagnosed with type Ⅲ BA during the operation. The same group of surgeons performed the operation throughout the research, employing consistent operative techniques and postoperative management. We adopted the modified extensive KP (8). The anatomic depth of the fibrous remnant was above the portal vein bifurcation to the outer edges of the portal vein (Figure 1B).

The resected fibrous remnants were oriented by the methods established in our previous research (6). Sections at the proximal end from the whole remnants were used to analyze neuronal plexuses and ductules. Sections with a thickness of 6 μm were prepared from paraffin-embedded biliary remnants at the cutting edge. The sections were further subjected to hematoxylin–eosin (HE) staining and acetylcholinesterase (AChE) immunochemical staining. Heat-induced antigen retrieval was performed using 10 mM sodium citrate (pH 6.0) buffer to expose target proteins. The sections were subjected to microwave treatment for 10 min. Following antigen retrieval, tissues were blocked in 3% bovine serum albumin–phosphate-buffered saline for 30 min at room temperature. Tissues were then probed at a dilution of 1:20 with the monoclonal AChE antibody (Ab2803, Abcam, Cambridge, MA, USA) overnight at 4°C in a humidified chamber. Tissues were washed extensively with PBS, and endogenous peroxidase activity was quenched with a peroxidase suppressor. The specific signals were detected using a commercial SP-0024 Histostain-Plus Kit (Bioss, Beijing, China). Colorimetric detection was performed using diaminobenzidine. Tissues were counterstained with hematoxylin and prepared for mounting. Two independent pathologists examined the results of HE staining and AChE staining of tissue specimens, focusing on the shape and extent of innervation in biliary remnants, AChE positivity, and the distribution and area of biliary ductules. The number and area of AChE-positive plexuses were measured and recorded.

In total, 67 patients exhibited stable bile drainage within 1 month after KP (Group A), while the other 20 patients showed no bile drainage during the follow-up periods (Group B). “Stable bile drainage” is defined as bile drainage that turns stool brown or dark green without the appearance of acholic stool. Patients with bile drainage were postoperatively treated with ursodeoxycholic acid capsules at 10 mg/kg bodyweight (three times a day) immediately after observation of stool containing bile. The other medications were given as previously described (6).

All patients were followed up for 24 months by phone. Various parameters including jaundice clearance at 3 or 6 months postoperatively, survival with the native liver at 1 and 2 years postoperatively, onset of cholangitis within 1 or 3 months postoperatively, total episodes of cholangitis onset, and preoperative/postoperative (at 1 month postoperatively) liver function markers (such as total bilirubin or TBIL, direct bilirubin or DBIL, *γ*-glutamyl transpeptidase or *γ*-GT, total bile acid or TBA, albumin or ALB, alanine aminotransferase or ALT, and aspartate aminotransferase or AST) were recorded and compared. The criterium for jaundice clearance was defined as total bilirubin <20 μmol/L.

SPSS19.0 software was used for statistical analysis. The counting data were expressed as relative composition ratios (%) or rates (%). Fisher's exact test was used to compare the jaundice clearance rate, incidence of cholangitis, and survival rate with the native liver. The results of liver function markers by blood tests were expressed as means ± standard deviations. All continuous data were compared using a two-way analysis of variance followed by the Mann–Whitney *U*-test. Spearman’s statistic was used to illustrate correlation. The 2-year survival with the native liver was assessed by the log rank test. The difference was considered statistically significant if the *p*-value was less than 0.05.

## Patient involvement

Patients were not involved in the design, conduct, reporting, or dissemination plans of our research.

## Results

### Pathomorphological characteristics of innervation in biliary remnants

Diffused fibrous connective tissue and varying numbers of proliferative biliary ductules in biliary remnants were seen in all cases. Small vessel dilation/hyperemia and diffused infiltration of inflammatory cells were also observed. In some cases, the growth of giant neuronal plexuses was observed (Figure 1C), and some proliferated ductules were also noted. No neuron existed in the bundles, but only thick nerve fiber bundles gathered in plexuses. The bundles were “wavy” or “vortex”-shaped (Figure 1D). Their morphology was similar to the exogenous neuronal plexuses in aganglionic colonic segments of patients with Hirschsprung's disease in our previous study (9). AChE in these plexuses was all positive, as shown by immunohistochemical staining (Figure 1E). However, in some other cases, no neuronal plexus was observed in biliary remnants (Figure 1F). In total, plexuses were seen in 21 biliary remnants, while they were absent in 66. In Group B, only one out of 20 had any plexus, whereas 20 out of 67 patients in Group A had plexuses (*p* = 0.034). Then, Group A was further divided into two subgroups, A1 and A2, according to the presence or absence of plexuses in biliary remnants.

### Clinical outcomes

In total, 77.0% (67/87) of patients had bile drainage within 1 month postoperatively. As shown in Table 1, there were 20 patients with postoperative bile drainage and plexuses in biliary remnants in Group A1, 47 patients with postoperative bile drainage but no plexuses seen in biliary remnants in Group A2, and 20 patients with neither bile drainage nor plexuses in Group B. There was no significant difference in gender distribution, mean age at operation, or preoperative liver function in the three groups (all *p*’s > 0.05). At 1 month postoperatively, in Group A1 and A2, except for the insignificant change in ALB levels, TBA (*p* = 0.022 and *p* = 0.153, respectively), TBIL (*p* = 0.000 and *p* = 0.000, respectively), DBIL (*p* = 0.000 and *p* = 0.000, respectively), ALT (*p* = 0.010 and *p* = 0.179, respectively), and AST (*p* = 0.000 and *p* = 0.000, respectively) levels appeared decreased to varying degrees compared with those preoperatively. However, the liver function in Group B worsened, especially the levels of TBIL (*p* = 0.022), DBIL (*p* = 0.052), and TBA (*p* = 0.006), which increased to varying degrees compared to preoperative levels.

**Table 1:** General information and liver function of patients.

		Group A1	Group A2	Group B		*p*-values	
					A1 vs. A2	B vs. A1	B vs. A2
Number of patients		20	47	20	N/A	N/A	N/A
Gender	Male	7	25	6	0.173	0.490	0.081
	Female	13	22	14			
Mean age at operation (days)		58.8 ± 15.0	64.9 ± 14.7	54.9 ± 11.0	0.063	0.499	0.067
TBIL	Preoperative	138.3 ± 47.2	155.8 ± 54.9	135.3 ± 42.1	0.233	0.931	0.211
	Postoperative	32.7 ± 32.8^a^	74.4 ± 84.8^a^	167.9 ± 53.3^b^	0.009	0.000	0.000
DBIL	Preoperative	108.9 ± 36.6	120.9 ± 40.9	113.7 ± 37.7	0.283	0.718	0.482
	Postoperative	29.8 ± 32.9^a^	59.4 ± 66.6^a^	141.0 ± 51.6	0.030	0.000	0.000
TBA	Preoperative	93.2 ± 27.1	97.3 ± 26.9	98.1 ± 29.0	0.507	0.602	0.867
	Postoperative	89.3 ± 94.9^a^	95.4 ± 70.0	125.3 ± 30.4^a^	0.206	0.003	0.002
ALB	Preoperative	39.4 ± 4.0	39.2 ± 3.5	39.8 ± 3.5	0.931	0.576	0.757
	Postoperative	37.8 ± 4.6	37.1 ± 4.0	36.8 ± 3.7	0.503	0.410	0.696
ALT	Preoperative	132.4 ± 77.0	126.9 ± 86.3	137.5 ± 94.9	0.349	0.941	0.277
	Postoperative	87.0 ± 55.8^a^	118.2 ± 102.8	153.8 ± 70.0	0.575	0.001	0.011
AST	Preoperative	184.8 ± 101.6	241.5 ± 171.8	197.5 ± 91.7	0.180	0.351	0.773
	Postoperative	82.9 ± 47.0^a^	136.0 ± 110.6^a^	212.3 ± 71.2	0.012	0.000	0.000

Group A1: postoperative bile drainage (+), nervous plexuses (+). Group A2: postoperative bile drainage (+), nervous plexuses (-). Group B: postoperative bile drainage (-), nervous plexuses (-).

^a^ *p *< 0.01 or ^b^*p *< 0.05 were considered statistically significant compared to preoperative levels.

### Jaundice clearance, cholangitis, and survival with the native liver

**Figure 2:**
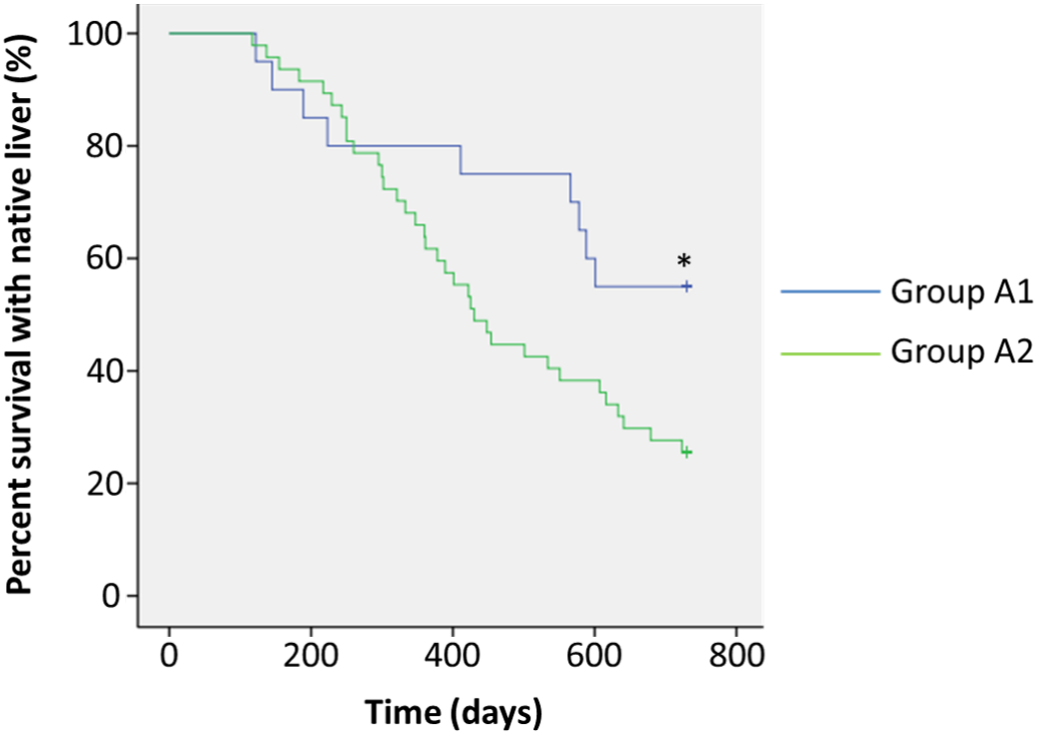
Analysis of survival with the native liver. Patients in Group A1 (*n* = 20) showed postoperative bile drainage and plexuses in biliary remnants (blue line). Patients in Group A2 (*n* = 47) showed postoperative bile drainage, but no plexuses were seen in biliary remnants (green line). *p < 0.05.

We next assessed jaundice clearance rates at 3 and 6 months postoperatively (Table 2). In Group A1, patients had the highest clearance rates at both time points (50% and 90%) compared to those in Groups A2 (19.1%, *p* = 0.010; 63.8%, *p* = 0.030) and B (10.0%, *p* = 0006; 0, *p* = 0.000). Episodes of early cholangitis onset within 1 month postoperatively appeared to be significantly fewer in Group A1 than in Group A2 (10.0% vs. 34.0%, *p* = 0.042). Within 3 months postoperatively, significantly fewer patients in Group A1 had onset of cholangitis than in Group A2 (55% vs. 78.7%, *p* = 0.048). During the follow-up period, 30% of patients in Group A1 had frequent (≥3 episodes) onset of cholangitis, which was significantly lower than in Group A2 (57.4%, *p* = 0.040). There was no significant difference between Group A1 and A2 in the overall survival with the native liver at 1 year postoperatively (80.0% vs. 61.7%, *p* = 0.144), whereas patients in these groups had significantly better survival with the native liver than those in Group B (30%, *p* = 0.001 and *p* = 0.017, respectively). However, a follow-up at 2 years postoperatively showed that patients’ survival rate with the native liver in Group A1 was significantly higher than that in Group A2 (*p* = 0.038) (Figure 2).

**Table 2:** Clinical outcomes.

		Group A1	Group A2	Group B		*p*-values	
					A1 vs. A2	B vs. A1	B vs. A2
Jaundice clearance at 3 months	Yes	10	9	2	0.010	0.006	0.355
	No	10	38	18			
Jaundice clearance at 6 months	Yes	18	30	0	0.030	0.000	0.000
	No	2	17	20			
Cholangitis within 1 month	Yes	2	16	0	0.042	0.147	0.003
	No	18	31	20			
Cholangitis within 3 months	Yes	11	37	2	0.048	0.002	0.000
	No	9	10	18			
Episodes of cholangitis onset	≥3	6	27	1	0.040	0.037	0.000
	<3	14	20	19			
Survival with the native liver at 1 year postoperatively	Yes	16	29	6	0.144	0.001	0.017
	No	4	18	14			

Group A1: postoperative bile drainage (+), nervous plexuses (+). Group A2: postoperative bile drainage (+), nervous plexuses (-). Group B: postoperative bile drainage (-), nervous plexuses (-).

### Correlations between biliary ductules, cholangitis, jaundice clearance, and neuronal plexuses

**Figure 3:**
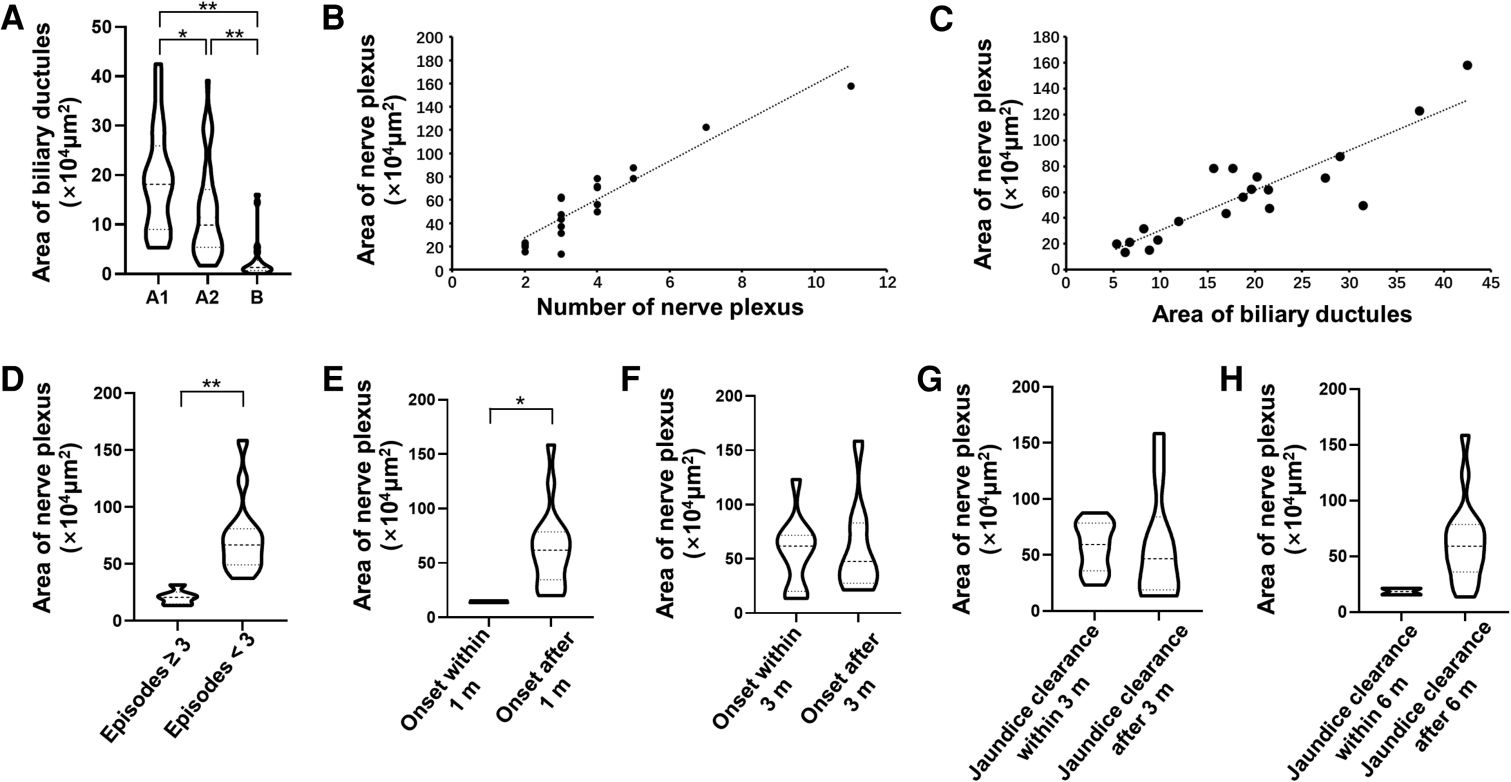
Analyses of ductules, plexuses, and postoperative prognosis of patients with biliary atresia. (**A**) Area of ductules in Groups A1, A2, and B. Group A1 had the largest area of ductules followed by Groups A2 and B. Group A1: postoperative bile drainage (+), nervous plexuses (+); Group A2: postoperative bile drainage (+), nervous plexuses (-); Group B: postoperative bile drainage (-), nervous plexuses (-). **p < 0.01 or *p < 0.05 were considered statistically significant compared to preoperative levels. (**B**) The number of plexuses was positively correlated with the area of plexuses. (**C**) The area of ductules was positively correlated with the area of plexuses. (**D**) Episodes of cholangitis and area of plexuses. Patients with ≥3 episodes had smaller areas than those with <3 episodes. **p < 0.01. (**E**) Early cholangitis and area of plexuses. Patients with early cholangitis had smaller areas of plexuses compared to those with cholangitis after 1 month postoperatively. *p < 0.05. (**F**) Area of plexuses and onset of cholangitis before or after 3 months postoperatively. Taking the 3-month postoperative period as the demarcation point, there was no significant difference in the area of the plexuses between the two subsets of patients. (**G**) Area of plexuses and jaundice clearance before or after 3 months postoperatively. Taking the 3-month postoperative period as the demarcation point, there was no significant difference in the area of the plexuses between the two subsets of patients. (**H**) Area of plexuses and jaundice clearance before or after 6 months postoperatively. Taking the 6-month postoperative period as the demarcation point, there was no significant difference in the area of the plexuses between the two subsets of patients.

We measured the areas of biliary ductules according to our previously established methods (6). We have previously examined the variation of innervation in remnants of 10 patients (from January 1, 2017, to September 30, 2017) by using continuous section; the shape and area of plexuses were insignificant within one remnant from the proximal to the distal end (data not shown). For the rest of the patients, we did not further compare the variation at different ends but only analyzed neuronal plexuses and ductules at the proximal end from the whole remnants. The area of ductules in Group A1 was significantly larger than that in Group A2 [(18.80 ± 10.53) × 10^4^ μm^2^ vs. (12.57 ± 9.37) × 10^4^ μm^2^, *p* = 0.011], whereas patients in Group B had the smallest areas [(18.80 ± 10.53) × 10^4^ μm^2^ vs. (2.92 ± 4.46) × 10^4^ μm^2^, *p* = 0.000; (12.57 ± 9.37) × 10^4^ μm^2^ vs. (2.92 ± 4.46) × 10^4^ μm^2^, *p* = 0.000] (Figure 3A). The number of plexuses ranged from 2 to 11 (average 3.85 ± 2.08), and the average area was (57.71 ± 36.83) × 10^4^ μm^2^. After regression analysis, the number of plexuses was found to be positively correlated with area (*R*^2 ^= 0.871, *p* = 0.000) (Figure 3B). In Group A1, a positive correlation was found between ductule areas and plexus areas (*R*^2 ^= 0.786, *p* = 0.000) (Figure 3C).

Patients in Group A1 with cholangitis episodes ≥3 or <3 were further subgrouped. We found that children with more episodes (median = 5.5) of cholangitis had significantly smaller areas of nerve plexuses than those with fewer episodes (median = 1.0) [(20.92 ± 6.43) × 10^4^ μm^2^ vs. (73.47 ± 32.77) × 10^4^ μm^2^, *p* = 0.000] (Figure 3D). Only two patients had early cholangitis in Group A1 within 1 month after surgery; the mean plexus area of the two cases was significantly smaller compared to the other 18 patients without early cholangitis onsets [(14.50 ± 1.27) × 10^4^ μm^2^ vs. (63.62 ± 36.44) × 10^4^ μm^2^, *p* = 0.0117] (Figure 3E). However, children with cholangitis onsets within 3 months after surgery had similar plexus areas to those without [(56.17 ± 32.36) × 10^4^ μm^2^ vs. (59.58 ± 43.64) × 10^4^ μm^2^, *p* = 0.941] (Figure 3F). At 3 months postoperatively, there was no significant difference in the areas of the nerve plexus between children who had jaundice clearance and those who did not [(57.54 ± 22.03) × 10^4^ μm^2^ vs. (57.87 ± 48.77) × 10^4^ μm^2^, *p* = 0.393] (Figure 3G). At 6 months postoperatively, although the plexus areas in children with jaundice clearance (*n* = 18) seemed larger than those in children without jaundice clearance (*n* = 2) [(62.07 ± 36.23) × 10^4^ μm^2^ vs. (18.40 ± 4.24) × 10^4^ μm^2^] (Figure 3H), the difference was not statistically significant (*p* = 0.063).

## Discussion

The porta hepatis in normal children is the major outlet of intrahepatic innervation, forming an anterior plexus around the hepatic artery and a posterior plexus around the portal vein and bile ducts (3). Instead of normal bile ducts in the porta hepatis, patients with BA only have biliary remnants, where the innervation appears, as shown in our current study. The morphological details revealed no neuron but only nerve fiber bundles in “thickened” plexuses. Because we cannot acquire normal bile ducts as control specimens, especially those of the same age, we do not know the characteristics of normal plexuses in 1–3-month-old children. The description of “thickened plexuses” is derived from our previous experience when examining exogenous neuronal plexuses in the aganglionic colons in patients with Hirschsprung's disease (9) and other previous studies (10). The plexus shape, cell shape, and positive AChE staining were three major similarities of plexuses between the two different diseases. Nevertheless, the plexuses in biliary remnants are gathered at marginal areas, while the plexuses in aganglionic colons are distributed around the colonic lumens. At least, based on the current evidence, we speculate that innervation inside the remnants is presented in some BA cases, yet we have not elucidated why not all remnants contain plexuses.

Cholinergic innervation was reported to derive from the vagus, with the major biological function of secreting acetylcholine and other neurotransmitters (11). As an important transmitter of the vagus nerve, acetylcholine can directly inhibit the activation of macrophages, thereby effectively reducing the release of a variety of pro-inflammatory factors, and it can act rapidly and persistently on specific tissues. Thus, it has a significant inhibitory effect on local and systemic inflammation (12). This recently discovered anti-inflammatory pathway associated with acetylcholine is known as the cholinergic anti-inflammatory pathway (13). Although the mechanism of cholinergic innervation in biliary remnants of children with BA remains unknown, in some experimental models of chronic hepatobiliary injury, such as the rat cirrhosis model induced by carbon tetrachloride, the cholinergic innervation in the damaged area was reported to be increased (14). Ungvary and Donath observed secondary changes in the distribution of intrahepatic nerves in cavies after ligation of the common bile duct, confirming a significant increase in nerve fibers in the porta hepatis (15). These early studies have shown the increase of innervation at various degrees in different types of liver injury, and they speculated that these changes could be associated with an increase in innate anti-inflammatory activities. It seems that in these above models, innervation is a consequence of inflammation or hepatic injury.

Viral infection is considered one of the possible theories for the pathogenesis of BA in neonates (16). Our serial studies previously showed the importance of viral antigens (17), innate immunity (18), and acquired immunity (19) in the development of experimental BA. Despite having not been fully elucidated, undeniable inflammation existed throughout the pathophysiological processes of BA and postoperative cholangitis, which is the most frequently encountered medical complication after KP. As known, postoperative cholangitis remains a significant challenge. It is closely related to a relatively delayed jaundice clearance (20) and may lead to increased early and late mortalities after KP for BA (21). We have also tried to explore the factors that may influence the onset frequency and severity of cholangitis and its therapeutic effect (6, 22, 23), but to date, doctors are still unable to link the onset of cholangitis and the morphological changes in biliary remnants. Through our current investigation, we claimed that the existence of cholinergic innervation at the transection where we cut the remnants may decrease the early onset of cholangitis and frequencies of recurrent cholangitis after KP.

Although it is yet unclear whether innervation is the consequence of inflammation, which serves as a feedback mechanism of bile duct injury, or the reason for ductule proliferation, which determines the number or area of ductules, a previous study has shown an extensive association between nerves and biliary ductules (24), and some other studies have shown that the activated cholinergic system may regulate ductal bile secretion (25). On the other hand, hepatic vagotomy is believed to inhibit cholangiocyte proliferation after bile duct ligation (26). These studies showed some positive roles of cholinergic innervation in the biliary system, but they did not show any direct relation between innervation and the growth of ductules. Researchers also believe that the loss of parenchymal nerve fibers is a consequence of worsening disease course in hepatic fibrotic diseases (3), and patients with BA lack intrahepatic bile duct innervation (7). Their research studies implicated that biliary innervation may form normally and then degrade as BA progresses. However, we found some connections between innervation and the number of ductules, which was crucial in postoperative bile flow drainage. As shown in Figures 1C,E, neuronal plexuses at the transection were clustered in the margin area of remnants, and the ductules distributed at lateral sides of remnants (6). Although there have been no studies showing mechanisms of how neuronal plexuses promote the hyperplasia of ductules or prevent ductules from damage, we found that the distribution of cholinergic plexuses depends on the number of biliary ductules (positive correlation referring to Figures 3B,C), indicating that the development or say destruction of cholinergic plexuses is subordinate to the capillary bile ducts. Although it is not a direct predictive factor that may give better bile drainage to patients with BA after KP, we need to pay attention to the potential research values of biliary innervation. Therefore, favorable bile flows may flush ductules persistently; thus, it may prevent the occurrence of reflux cholangitis.

Finally, we aimed to analyze the influence of the number/area of neuronal plexuses on the onset of cholangitis in patients with cholinergic innervation. A smaller area of plexuses was correlated to more episodes of cholangitis and a greater possibility of early cholangitis onset (within 1 month). Theoretically, increased cholinergic innervation should mean a potential increase in acetylcholine activity, which plays a potential anti-inflammatory role (13). However, within 3 months, patients with different areas of plexuses showed no statistical difference in the episodes of cholangitis, nor did the time of jaundice clearance between them show any differences. Owing to that, we cannot observe the change in the hepatojejunal anastomosis during the following months after surgery, and we are unable to analyze the change patterns of neuronal plexuses in remnants at the transection in patients after surgery. Nevertheless, we speculate that there may be certain aspects of morphological or functional changes in innervation after surgery, similar to the report by Iwami et al. showing that biliary innervation may form normally and then degrade as BA progresses (7). Thus, after months of bile drainage, innervation at the transection may have become similar in patients who previously had different levels of innervation when undergoing KP. Therefore, the difference in anti-inflammatory effect, which exists in the early days after KP, may no longer exist in these patients several months postoperatively.

## Conclusion

Cholinergic innervation at the transection of biliary remnants may help reduce the onset of cholangitis and lead to better and earlier jaundice clearance. Innervation may lead to better bile drainage by affecting the areas of biliary ductules or by increasing acetylcholine activity. Children with larger plexus areas are less likely to have an early episode of cholangitis. Based on our current findings, we believe that it is worth exploring the roles of acetylcholine and its associated anti-inflammatory pathway during the development of BA and in postoperative cholangitis in the future.

## Data Availability

The original contributions presented in the study are included in the article/Supplementary Material; further inquiries can be directed to the corresponding author.
